# Single-Cell RNA Sequencing Reveals LEF1-Driven Wnt Pathway Activation as a Shared Oncogenic Program in Hepatoblastoma and Medulloblastoma

**DOI:** 10.3390/curroncol32010035

**Published:** 2025-01-09

**Authors:** Christophe Desterke, Yuanji Fu, Jenny Bonifacio-Mundaca, Claudia Monge, Pascal Pineau, Jorge Mata-Garrido, Raquel Francés

**Affiliations:** 1 Faculté de Médecine du Kremlin Bicêtre, Université Paris-Saclay, INSERM UMRS-1310, 94805 Villejuif, France; christophe.desterke@inserm.fr; 2 Institut Necker Enfants Malades, INSERM, CNRS, Université Paris Cité, 75015 Paris, France; yuanji.fu@inserm.fr; 3 National Tumor Bank, Department of Pathology, National Institute of Neoplastic Diseases, Lima 15024, Peru; jenny.bonifacio@upch.pe; 4 Institut Pasteur, Université Paris Cité, Unité Organisation Nucléaire et Oncogenèse, INSERM U993, 75015 Paris, France; claudia.monge@pasteur.fr (C.M.); pascal.pineau@pasteur.fr (P.P.); 5 Energy & Memory, Brain Plasticity Unit, CNRS, ESPCI Paris, PSL Research University, 75006 Paris, France

**Keywords:** LEF1, WNT, pluripotency, pediatric cancer, hepatoblastoma, medulloblastoma, genome binding occupancy, scRNA sequencing, omics

## Abstract

(1) Background: Hepatoblastoma and medulloblastoma are two types of pediatric tumors with embryonic origins. Both tumor types can exhibit genetic alterations that affect the β-catenin and Wnt pathways; (2) Materials and Methods: This study used bioinformatics and integrative analysis of multi-omics data at both the tumor and single-cell levels to investigate two distinct pediatric tumors: medulloblastoma and hepatoblastoma; (3) Results: The cross-transcriptome analysis revealed a commonly regulated expression signature between hepatoblastoma and medulloblastoma tumors. Among the commonly upregulated genes, the transcription factor LEF1 was significantly expressed in both tumor types. In medulloblastoma, LEF1 upregulation is associated with the WNT-subtype. The analysis of LEF1 genome binding occupancy in H1 embryonic stem cells identified 141 LEF1 proximal targets activated in WNT medulloblastoma, 13 of which are involved in Wnt pathway regulation: *RNF43*, *LEF1*, *NKD1*, *AXIN2*, *DKK4*, *DKK1*, *LGR6*, *FGFR2*, *NXN*, *TCF7L1*, *STK3*, *YAP1*, and *NFATC4*. The ROC curve analysis of the combined expression of these 13 WNT-related LEF1 targets yielded an area under the curve (AUC) of 1.00, indicating 100% specificity and sensitivity for predicting the WNT subtype in the PBTA medulloblastoma cohort. An expression score based on these 13 WNT-LEF1 targets accurately predicted the WNT subtype in two independent medulloblastoma transcriptome cohorts. At the single-cell level, the WNT-LEF1 expression score was exclusively positive in WNT-medulloblastoma tumor cells. This WNT-LEF1-dependent signature was also confirmed as activated in the hepatoblastoma tumor transcriptome. At the single-cell level, the WNT-LEF1 expression score was higher in tumor cells from both human hepatoblastoma samples and a hepatoblastoma patient-derived xenotransplant model; (4) Discussion: This study uncovered a shared transcriptional activation of a LEF1-dependent embryonic program, which orchestrates the regulation of the Wnt signaling pathway in tumor cells from both hepatoblastoma and medulloblastoma.

## 1. Introduction

Hepatoblastoma and medulloblastoma are two rare, aggressive pediatric cancers of embryonic origin that present unique challenges for treatment due to their underlying genetic complexity and shared developmental pathways [1,2]. These tumors frequently involve dysregulation in the Wnt/β-catenin signaling pathway, which is essential for embryonic development and the maintenance of stem cell populations [3,4,5]. According to the World Health Organization (WHO) classification, hepatoblastomas are categorized into distinct subtypes based on their histological composition, including purely epithelial forms (fetal, embryonal, and small-cell undifferentiated) and mixed epithelial and mesenchymal types, with certain subtypes linked to worse prognoses [6]. Mutations in CTNNB1, which encodes β-catenin, are commonly observed in both hepatoblastoma and certain medulloblastoma subtypes, particularly those classified as WNT subtypes, highlighting a shared oncogenic mechanism that implicates Wnt signaling in the progression and characteristics of these cancers [7,8].

Central to Wnt pathway activity is the transcription factor LEF1, part of the TCF/LEF family, which serves as a key mediator of β-catenin signaling (Supplemental Figure S1) [9,10]. LEF1 drives the expression of target genes associated with cellular proliferation, differentiation, and survival, functions that are often hijacked in cancer to promote tumor growth [11,12]. Recent findings suggest that LEF1’s influence extends beyond traditional Wnt pathway signaling, impacting metabolic and epigenetic landscapes within tumors [13]. These roles, however, remain incompletely understood, particularly in the context of a shared LEF1-mediated transcriptional program across hepatoblastoma and medulloblastoma.

This study aims to bridge this gap by investigating the common transcriptional and molecular features driven by LEF1 in both hepatoblastoma and WNT-subtype medulloblastoma. Through integrative bioinformatics and multi-omics analyses at both the tumor and single-cell levels, we identify a robust WNT-LEF1 gene expression signature enriched in tumor cells from both cancers. This signature, comprising critical regulatory genes, highlights a conserved embryonic transcriptional program likely contributing to tumor progression and therapeutic resistance. By elucidating the LEF1-dependent mechanisms within the Wnt pathway, our findings provide new insights into the molecular underpinnings of these two pediatric tumors and reveal potential targets for therapeutic intervention that leverage this shared oncogenic axis.

## 2. Materials and Methods

### 2.1. Public Transcriptome Datasets

#### 2.1.1. MB-PBTA (MB, Open PBTA)

The Open Pediatric Brain Tumor Atlas [14]: RNA-sequencing data for medulloblastoma from the PBTA were downloaded from the Pediatric cBioPortal website, part of the “Open Pediatric Cancer Project” consortium [15], at the following address: https://pedcbioportal.kidsfirstdrc.org/ (accessed on 6 November 2024). After filtration and clinical annotation, this transcriptomic medulloblastoma cohort consisted of 254 tumor samples, with most samples from male patients (61%) and primary tumors (61%). These tumors were distributed among distinct molecular subtypes: WNT (n = 21), SHH (n = 74), G3 (n = 60), and G4 (n = 99) (Table 1). RNA-sequencing counts were transformed into pseudocounts using a (log2 + 1) data transformation for downstream analyses.

#### 2.1.2. GSE37418 (MB)

Normalized data matrices and annotations (GPL570-[HG-U133_Plus_2] Affymetrix Human Genome U133 Plus 2.0 Array) from dataset GSE37418 [17] were downloaded from the Gene Expression Omnibus (GEO) website [18] using the GEOquery R package version 2.70.0 [19] in the R software environment version 4.3.3. This transcriptomic cohort of medulloblastoma tumors included 74 tumor samples, with the majority from male patients (71%) and a mean age of 99 months. Most patients were of white ethnicity (57%), followed by Hispanic ethnicity (18%). Regarding molecular subtypes, this cohort consisted of 39 G4 samples, 8 WNT samples, 11 SHH samples, and 16 G3 samples (Table 2).

#### 2.1.3. GSE44971 (Normal Cerebellum)

Normalized data matrices and annotations (GPL570-[HG-U133_Plus_2] Affymetrix Human Genome U133 Plus 2.0 Array) from dataset GSE44971 [20] were downloaded from the GEO website [18] using the GEOquery R package version 2.70.0 [19] in the R software environment version 4.3.3. This cohort comprised samples of normal cerebellar tissue.

#### 2.1.4. GSE104766 (HB and Normal Liver)

RNA-sequencing normalized counts (Illumina HiSeq 2500, Homo sapiens) were downloaded from the GEO NCBI website at https://www.ncbi.nlm.nih.gov/geo/query/acc.cgi?acc=GSE104766 (accessed on 6 November 2024). This cohort included 30 hepatoblastoma tumor samples and 30 normal liver samples. No associated clinical data were provided for this cohort [21].

#### 2.1.5. GSE131329 (HB)

This RNA-seq transcriptomic cohort comprised 14 samples of noncancerous liver tissue and 53 samples of hepatoblastoma tumors [22]. Of the pathological samples, 30 were histologically well differentiated, and 20 were poorly differentiated. The mean age of the patients at the time of sampling was 27 months. Each PRETEXT stage (1–4) was well balanced among the study samples, and the majority of samples exhibited β-catenin genomic alterations (Table 3).

### 2.2. Public Single-Cell Transcriptome Datasets

#### 2.2.1. scRNA-Seq Medulloblastoma

The GSE155446 dataset comprised single-cell RNA-sequencing data from 28 pediatric medulloblastoma samples, stratified into the four medulloblastoma subtypes: SHH, WNT, G3, and G4. For these experiments, libraries were prepared using Chromium Single Cell V2 and V3 Chemistry Library Kits (10× Genomics). Barcoded cDNA was sequenced on an Illumina NovaSeq 6000, achieving 50,000 reads per cell. Data were demultiplexed using CellRanger (10× Genomics) [23]. Batch-corrected original raw counts were integrated into a single-cell object using Seurat version 5.1.0. The standard Seurat pipeline, including NormalizeData, FindVariableFeatures, ScaleData, and RunPCA, was applied to the single-cell object [24]. A single-cell expression score was computed using a 13-gene signature with the AddModuleScore function in Seurat.

#### 2.2.2. scRNA-Seq Hepatoblastoma

Single-cell RNA-sequencing data from the GEO dataset GSE180665 [25] were downloaded from the following web address: https://www.ncbi.nlm.nih.gov/geo/query/acc.cgi?acc=GSE180665 (accessed on 1 August 2024). This dataset included seven scRNA-seq experiments performed on three hepatoblastoma tumors, two patient-derived xenotransplantation (PDX) models, and two adjacent liver tissue samples. Raw counts in H5AD format were converted into a single-cell experiment object using the zellkonverter R-Bioconductor package [26]. The original cell annotation was added to the metadata of the scRNA-seq object after its conversion into a Seurat object to perform harmony batch correction during sample integration [24].

### 2.3. ChIP-Sequencing in Human Pluripotent H1 Cell Line

To characterize LEF1 chromatin binding occupancy in humans at the embryonic stage within the context of the bivalent promoter program [27], different ChIP-sequencing datasets performed on H1 human pluripotent stem cells were accessed from the Gene Expression Omnibus (GEO) database [18]:

GSM1579343: LEF1 untreated; Homo sapiens with SRA alias SRR1745491;
GSM1579344: LEF1 Wnt3a; Homo sapiens with SRA alias SRR1745492;
GSM1693959: Input H1; Homo sapiens with SRA alias SRR2037029;
GSM1579348: H3K27ac Wnt3a; Homo sapiens with SRA alias SRR1745496;
GSM1579350: H3K27me3 Wnt3a; Homo sapiens with SRA alias SRR1745498.

These experiments were conducted by the Jones Lab in San Diego, California [28]. For ChIP-seq, DNA was extracted using the QIAquick PCR extraction kit (cat 28106, Qiagen, Venlo, The Netherlands). Single-end reads were sequenced on an Illumina HiSeq 2500. The ChIP-sequencing data were downloaded in FASTQ format from the NCBI database using the fastq-dump command from the SRA Toolkit (version 3.1.1) [29].

### 2.4. Transcriptome Cross Normalization for Common Signature Between Hepatoblastoma and Medulloblastoma Tumors

Transcriptome analyses were conducted in the R software environment (version 4.3.3). To investigate common regulatory mechanisms in hepatoblastoma and medulloblastoma, a cross-normalized matrix was constructed by merging distinct transcriptome datasets: GSE37418, comprising medulloblastoma tumor samples; GSE44971, comprising normal cerebellum controls; and GSE104766, comprising hepatoblastoma tumors and normal liver samples.

To account for experimental batch effects, cross-normalization was performed using the Combat function from the SVA R package (version 3.50.0) [30]. Following the Combat processing step, quantile normalization was applied to reduce noise between samples using the PreprocessCore R package (version 1.64.0) [31].

For downstream analyses, the LIMMA (Linear Models for Microarray Data) R package (version 3.58.1) was employed to identify differentially expressed genes between tumor samples (hepatoblastoma and medulloblastoma) and their respective controls (normal liver and normal cerebellum) [32]. Functional enrichment analyses of significant genes were conducted using the clusterProfiler R package (version 4.10.1) [33,34] with Gene Ontology Molecular Function and Biological Process databases [35].

### 2.5. ChIP-Sequencing Analyses

Chromatin immunoprecipitation sequencing (ChIP-seq) experiments were analyzed following the recommended guidelines of the ENCODE consortium [36]. Downloaded FASTQ files were assessed for quality using FastQC (version 0.74). FASTQ files were trimmed with Trimmomatic (version 0.39) [37], and the quality of the trimmed reads was subsequently evaluated with FastQC.

Reads were aligned to the hg38 human genome using Bowtie2 (version 2.5.3) in very sensitive end-to-end mode [38]. BAM files were sorted using SAMtools (version 1.19.2) [39] and filtered for mapping quality scores greater than 30 with BamTools (version 2.5.2) [40]. Narrow peaks representing LEF1 genome binding in H1 cells stimulated with WNT3A were called using the MACS2 peak caller (version 2.2.9.1) [41,42], with H1 input chromatin serving as the background control [43].

MACS2-generated narrow peaks were annotated with HOMER (version 4.11) [44] on the hg38 genome, using the GencodeV46 annotation file [45]. Promoter-annotated regions from the resulting BED file were filtered for downstream analysis. BigWig files were generated using the bamCoverage command from deeptools (version 3.5.4) [46]. Genomic heatmaps of promoter regions were created using the double commands computeMatrix and plotHeatmap from deepTools.

Functional enrichment analyses of significant genes were conducted using the clusterProfiler R package (version 4.10.1) [33,34] with Gene Ontology Molecular Function and Biological Process databases [35].

### 2.6. WNT-LEF1 Expression Score

Using the expression of 13 WNT-LEF1 target genes in the medulloblastoma PBTA cohort, univariate logistic regression analyses were conducted using the logitloop R package (version 1.0.0), available at https://github.com/cdesterke/logitloop (accessed on 13 November 2024). The binomial status of the WNT subtype was used as the outcome variable for the regression analyses.

A WNT-LEF1 expression score was subsequently calculated by summing the expression levels of the target genes weighted by the binomial β-coefficients obtained from the univariate analyses. The optimal cutpoint for the expression score was determined using the cutpointr R package (version 1.1.2) [47].

### 2.7. ElasticNet Modeling on WNT-LEF1 Expression Signature

The expression levels of the 13 WNT-LEF1 target genes were extracted from medulloblastoma tumors and combined with the corresponding WNT subtype status as metadata. After splitting the data into training and validation sets in a 0.6/0.4 ratio, an ElasticNet model (with tumor cell status as the binary outcome) was tuned for alpha and lambda parameters using the caret R package (version 6.0-94) [48]. The final ElasticNet model was fitted with the optimal alpha parameter (alpha = 0.1) using the glmnet R package (version 4.1-8) [49].

## 3. Results

### 3.1. LEF1 Upregulation as a Shared Molecular Feature in Hepatoblastoma and Medulloblastoma

The WNT subgroup of medulloblastoma is characterized by its distinct molecular profile, driven primarily by aberrations in the WNT signaling pathway, such as mutations in *CTNNB1*. Previous studies suggest that the WNT subgroup likely originates from progenitor cells in the lower rhombic lip or dorsal brainstem, regions involved in embryonic development [50]. This aligns with the unique transcriptional and epigenetic landscape observed in WNT tumors, further distinguishing them from other medulloblastoma subgroups. However, the precise cell of origin remains a topic of active investigation, underscoring the need for deeper developmental insights.

Hepatoblastoma and medulloblastoma are two types of pediatric tumors with embryonic origins. Tumor cells in these two types frequently exhibit genetic alterations in WNT pathway genes, particularly involving mutations in *CTNNB1*. Based on these shared characteristics, we constructed a combined transcriptome dataset that includes medulloblastoma and hepatoblastoma tumor samples, along with control samples from the respective tissues of origin (cerebellum for medulloblastoma and adjacent healthy liver for hepatoblastoma).

**Figure 1:**
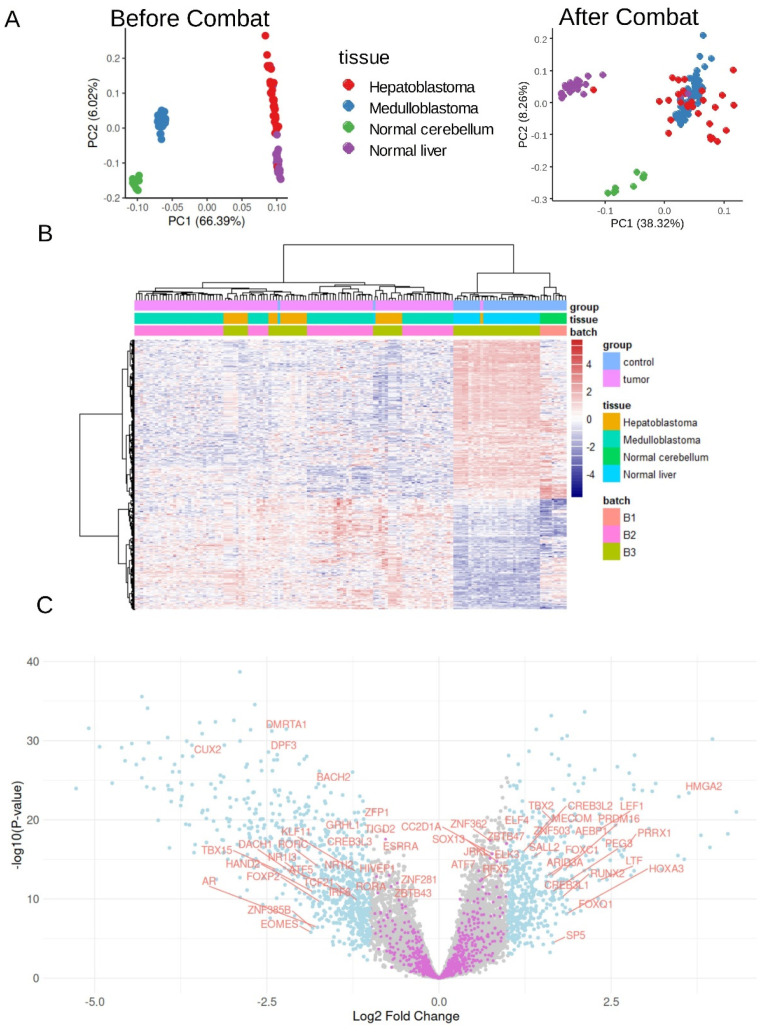
Common transcriptional regulation between hepatoblastoma and medulloblastoma tumors: cross-combat normalization between GSE37418, GSE44971, and GSE104766 datasets. (**A**) Principal component analysis (PCA) performed on whole transcriptome before and after combat batch correction (HB: hepatoblastoma and MB: medulloblastoma) and control samples (normal cerebellum and normal liver). (**B**) Unsupervised clustering (Euclidean distances) based on the common HB-MB signature (1259 DEGs). (**C**) Volcano plot highlighting the significant transcription factors (light blue and annotated) regulated between tumors (HB and MB) and normal tissues (cerebellum and liver).

To conduct this analysis, we assembled data from the GSE37418 (medulloblastoma), GSE44971 (cerebellum control), and GSE104766 (normal liver and hepatoblastoma) datasets, normalizing them in a common matrix using the Combat algorithm to adjust for batch effects (Supplemental Figure S2a). The heterogeneity in Combat density plots across samples (Supplemental Figure S2b) was reduced by applying quantile post-Combat normalization (Supplemental Figure S2c). Unsupervised principal component analysis (PCA) of the fully normalized transcriptome data confirmed that samples from different batches were well integrated (Supplemental Figure S3A and Figure 1A). Additionally, this analysis clearly distinguished tumor samples (medulloblastoma and hepatoblastoma) from normal controls (cerebellum and liver) (Supplemental Figure S3B), suggesting a shared transcriptional expression signature between these tumors.

To identify this common transcriptional signature, we performed a supervised differential expression analysis between tumor and control samples using the LIMMA algorithm. With a significance threshold of |log2 fold change| ≥ 1 and an FDR-adjusted *p*-value < 0.05, we identified 1259 differentially expressed genes (DEGs) significantly regulated in both hepatoblastoma and medulloblastoma compared to their respective controls (volcano plot in Supplemental Figure S3C; list of DEGs in Supplemental Table S1). Unsupervised clustering of these 1259 DEGs successfully stratified tumor samples from controls using Euclidean distances and the Ward.D2 method (Figure 1B).

Functional enrichment analysis using the Gene Ontology Molecular Function (GO-MF) database revealed that the 513 upregulated genes in tumors were predominantly associated with extracellular matrix interactions, including structural components (e.g., collagens), integrin binding, collagen binding, and growth factor binding (Supplemental Figure S4a,b). Conversely, the analysis of the 746 downregulated genes in tumors highlighted a significant enrichment in vitamin-interacting molecules and enzymes with aromatase activity, such as cytochrome P450 family members (Supplemental Figure S5a,b).

Using Toronto’s database of human transcription factors [51], we projected transcription factor identifiers onto the volcano plot (Figure 1C) to examine their regulation in tumors. Notable upregulated transcription factors included HMGA2, TBX2, CREB3L2, and LEF1 (Figure 1C and Supplemental Table S2). Among these, ChIP-sequencing data on LEF1 binding in human embryonic H1 stem cells have been previously conducted [28]. This epigenetic information allows us to investigate the LEF1-dependent transcriptional program that operates during human embryonic development.

### 3.2. WNT3A Stimulation Activates LEF1 Chromatin Binding Program with H3K27ac Co-Occupancy in H1 Embryonic Cells

LEF1 ChIP-sequencing experiment conducted on H1 pluripotent human stem cells after WNT3A stimulation was aligned to the hg38 human genome using the Bowtie2 algorithm. Similar ChIP-sequencing experiments were also performed for H1 input chromatin and LEF1 in H1 cells without WNT3A stimulation. Additionally, the bivalency of pluripotent promoter regions was examined using ChIP-sequencing for histone marks H3K27me3 (a repressive mark) and H3K27ac (an active mark) following the same pipeline. Narrow peak calling for regions with high-quality mapped reads (Q > 30) was carried out using the MACS2 algorithm, with H1 input chromatin as a control for peak calling in LEF1-H1-WNT3A ChIP-seq. This analysis identified 11,436 enriched genomic regions.

**Figure 2:**
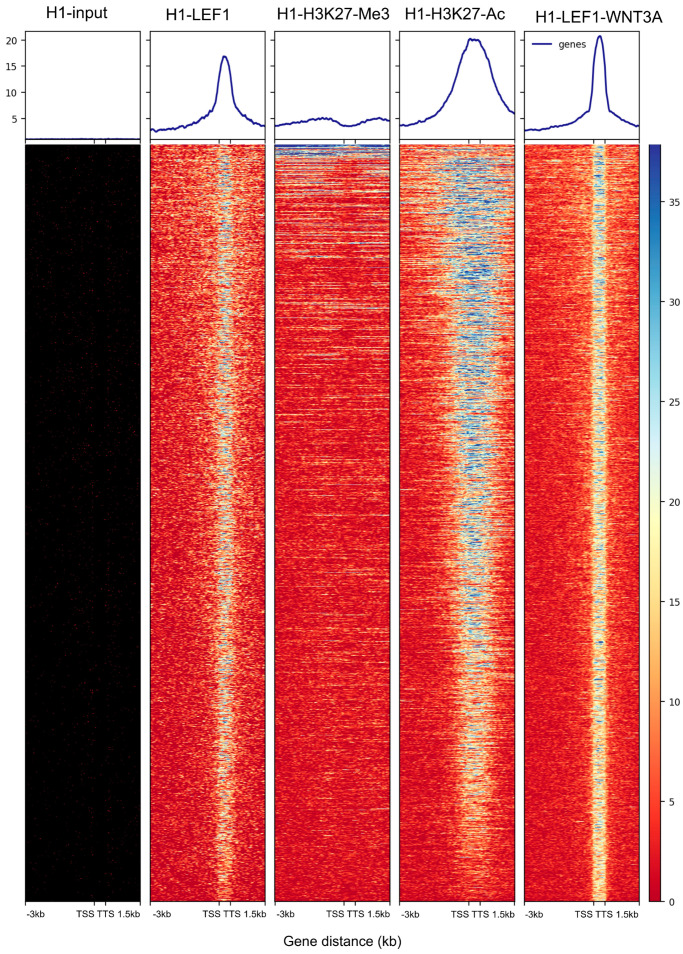
Embryonic program of LEF1 genome binding occupancy after WNT3A stimulation: genomic heatmap illustrating the enrichment of genomic intervals (hg38) for LEF1 binding in H1 human embryonic stem cell chromatin after WNT3A stimulation. From left to right, respective enrichment was shown for ChIP-seq of H1 input chromatin, LEF1 in unstimulated H1 cells, H3 histone lysine 27 trimethylation (repressive mark), H3 histone lysine 27 acetylation (active mark), and LEF1 in WNT3A-stimulated H1 cells. (TSS: transcription start site and TTS: transcription termination site).

After annotating peaks with Homer, 2039 were found to be in proximal promoter regions (Supplemental Table S3). For these 2039 promoter regions, a genomic matrix was generated using deeptools, centered around Transcription Start Sites (TSSs) on the hg38 genome, extending 3 kb upstream and 1.5 kb downstream. This analysis included five ChIP-sequencing experiments: H1 input, H1-LEF1, H1 H3K27me3, H1 H3K27ac, and H1-LEF1-WNT3A. The resulting genomic heatmap (Figure 2) confirmed the presence of these promoter regions in the H1-LEF1-WNT3A ChIP-seq experiment, with no corresponding peaks in the H1 input chromatin ChIP-seq. These enrichments were also observed in the H1-LEF1 ChIP-seq experiment, though with reduced intensity compared to the WNT3A stimulation condition. Notably, these promoter regions largely coincided with active histone marks, specifically H3K27ac (Figure 2). Through this analysis, an active LEF1 embryonic chromatin binding program was identified in H1 stem cells upon stimulation with the WNT pathway ligand WNT3A.

### 3.3. LEF1 Embryonic Transcriptional Network Is a Hallmark of WNT-Subtype Medulloblastoma

**Figure 3:**
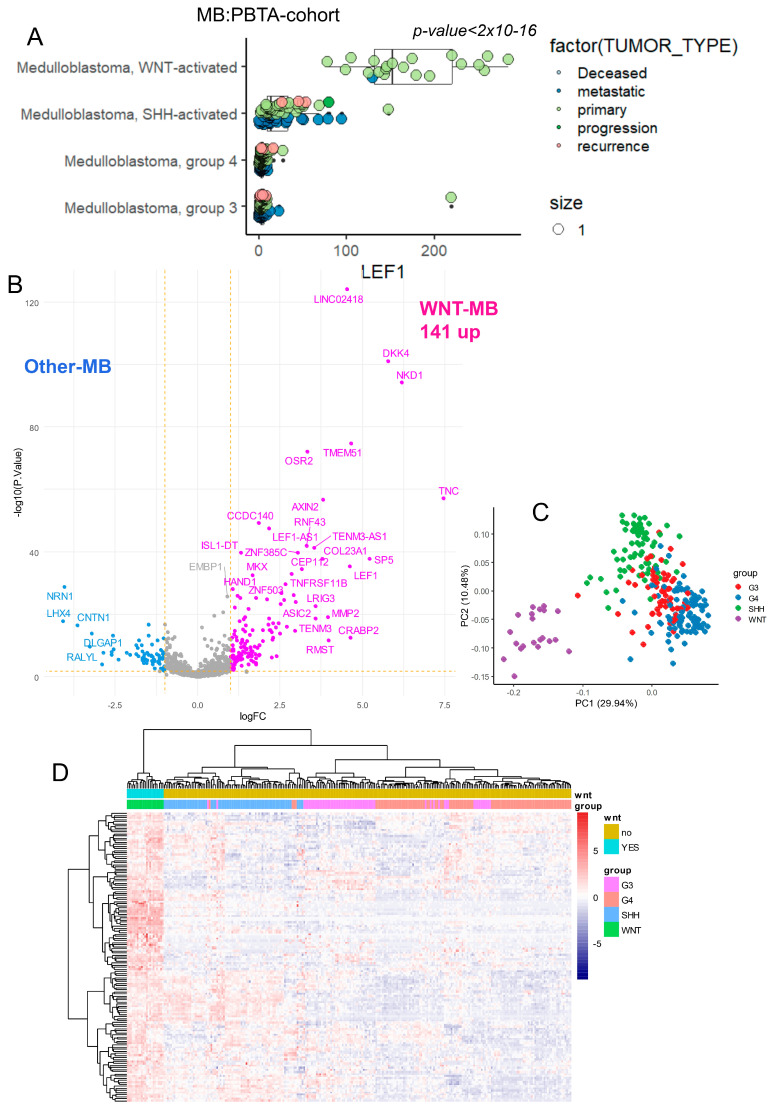
LEF1 embryonic binding program predicts the WNT subtype in the medulloblastoma transcriptome: integration of the LEF1 binding program in the MB-PBTA transcriptome cohort. (**A**) Expression of LEF1 in the medulloblastoma transcriptome of the PBTA cohort stratified by medulloblastoma subtypes: WNT, SHH, G3, and G4. (**B**) Volcano plot of differentially expressed genes (with embryonic LEF1 binding) between the WNT-MB subtype and other MB samples. (**C**) Principal component analysis (PCA) performed on the 141 LEF1 embryonic targets overexpressed in the WNT-MB subtype. (**D**) Unsupervised clustering (Euclidean distances) based on the expression of the 141 LEF1 embryonic targets overexpressed in the WNT-MB subtype.

In embryonic medulloblastoma, heterogeneity in WNT pathway activation has been characterized through transcriptome analysis [15,17]. The Pediatric Brain Tumor Atlas (PBTA) cohort [14] was filtered to include RNA-sequencing data from medulloblastoma samples classified by molecular subtypes: WNT, SHH, Group 3 (G3), and Group 4 (G4) (n = 254; Table 1). LEF1 expression was analyzed according to the molecular subtype classification within the PBTA cohort (Figure 3A). LEF1 was found to be significantly increased in the transcriptomes of WNT subtype medulloblastomas compared to other subtypes (ANOVA *p* ≤ 2 × 10^−16^), predominantly in samples categorized as primary tumors (Figure 3A).

LEF1 target genes, identified through ChIP-sequencing as 2039 promoter regions (Supplemental Table S3), were mapped onto the transcriptome data of medulloblastoma samples in the PBTA cohort. A supervised differential expression analysis was performed between WNT subtype tumors and other molecular subtypes to assess the regulation of LEF1 target genes. Using a significance threshold of |log2 fold change| ≥ 1 and FDR-adjusted *p*-value < 0.05, 207 DEGs were identified, with the majority (n = 141) being overexpressed in WNT subtype tumors compared to other medulloblastoma subtypes (Figure 3B; Supplemental Table S4). Principal component analysis (PCA) based on the expression of these 141 LEF1 target genes effectively stratified WNT subtype tumors from other medulloblastoma subtypes (Figure 3C). This stratification was further validated using unsupervised hierarchical clustering with Euclidean distances and the Ward.D2 method (Figure 3D).

**Figure 4:**
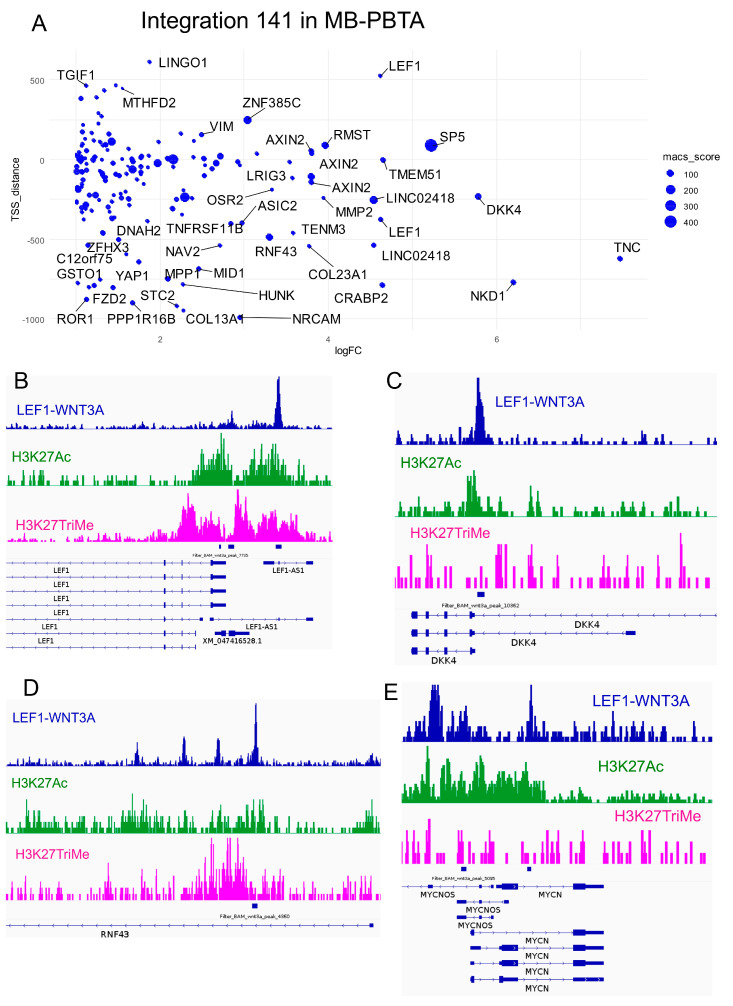
Heterogeneity of LEF1-dependent embryonic promoters for targets activated during WNT medulloblastoma: integration into the MB-PBTA cohort. (**A**) Scatterplot of the integrative analysis representing ChIP-seq peak distances from TSS versus log2 fold changes in the WNT-MB transcriptome. Dot sizes represent peak scores from the MACS2 algorithm for LEF1-H1-WNT3A ChIP-seq data. (**B**) Promoter visualization for the LEF1 and LEF1-AS loci. (**C**) Promoter visualization for the DKK4 locus. (**D**) Promoter visualization for the RNF43 locus. (**E**) Promoter visualization for the MYCN locus.

Functional enrichment analysis (Gene Ontology Biological Process [GO-BP] database) of the 141 LEF1 target genes highlighted their roles in mesenchymal development, embryonic organ development, and WNT pathway regulation (Supplemental Figure S6a, Figure 4A and Supplemental Table S5). Notably, bivalent histone marks (positive for both H3K27ac and H3K27me3) were detected at the genomic loci of LEF1/LEF1-AS1 (Figure 4B) and RNF43 (a WNT pathway component and LEF1 target; Figure 4D). Active histone marks (H3K27ac) were also found at the promoters of DKK4 (WNT pathway regulator and LEF1 target; Figure 4C) and MYCN (a LEF1 target; Figure 4E).

**Figure 5:**
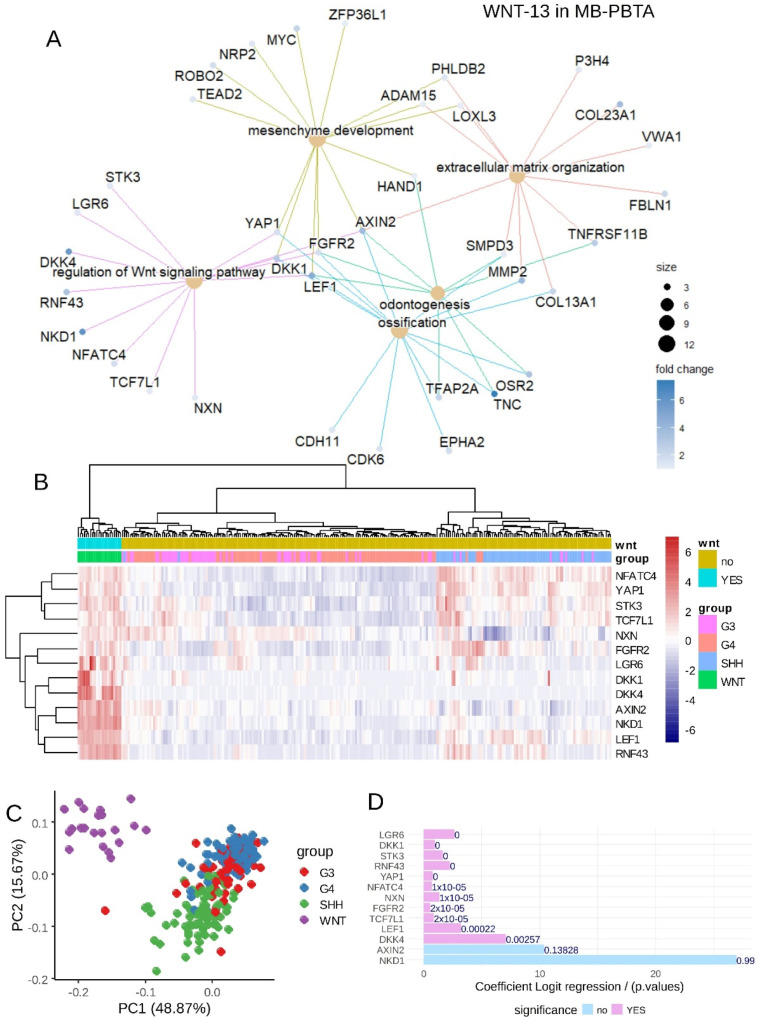
WNT signaling LEF1-dependent signature in medulloblastoma: MB-PBTA cohort. (**A**) Functional enrichment network based on LEF1 targets activated during the WNT-medulloblastoma subtype using the Gene Ontology Biological Process (GO-BP) database. (**B**) Unsupervised clustering (Euclidean distances) of the WNT-program LEF1-dependent genes in medulloblastoma tumors. (**C**) Principal component analysis (PCA) of the WNT-program LEF1-dependent genes in medulloblastoma tumors. (**D**) Univariate binomial regression analysis of the 13 WNT signaling target genes of LEF1 during medulloblastoma (outcome: WNT subtype).

Among the LEF1 targets upregulated in WNT medulloblastoma, 13 genes were identified as regulators of the WNT pathway (Figure 5A). These included RNF43 (ring finger protein 43), LEF1 (lymphoid enhancer binding factor 1), NKD1 (NKD inhibitor of WNT signaling pathway 1), AXIN2 (axin 2), DKK4 (dickkopf WNT signaling pathway inhibitor 4), DKK1 (dickkopf WNT signaling pathway inhibitor 1), LGR6 (leucine-rich repeat-containing G protein-coupled receptor 6), FGFR2 (fibroblast growth factor receptor 2), NXN (nucleoredoxin), TCF7L1 (transcription factor 7 like 1), STK3 (serine/threonine kinase 3), YAP1 (Yes1 associated transcriptional regulator), and NFATC4 (nuclear factor of activated T cells 4). Using the expression profiles of these 13 WNT-related LEF1 target genes, unsupervised clustering (Euclidean distances and the Ward.D2 method) effectively stratified WNT-medulloblastoma samples from some samples in other medulloblastoma subtypes in the PBTA cohort (Figure 5B). This stratification was corroborated by PCA (Figure 5C), and univariate binomial analysis with WNT-medulloblastoma status as the outcome confirmed significant associations for 11 of the 13 genes (Figure 5D).

The ROC curve analysis of the combined expression of these 13 WNT-related LEF1 targets yielded an area under the curve (AUC) of 1.00, indicating 100% specificity and sensitivity for predicting the WNT subtype in the PBTA medulloblastoma cohort (Supplemental Figure S6b). Based on the expression and binomial β-coefficients of the 13 WNT-LEF1 genes (Figure 5D), a WNT-LEF1 expression score was computed for medulloblastoma samples in the PBTA cohort. This analysis identified 21 samples with high WNT-LEF1 expression scores, compared to 233 remaining samples with lower scores (Table 1). Between the high- and low-score groups, no significant gender bias was observed (*p* = 1.00). However, high-score samples were primarily categorized as primary tumors (*p* = 0.023), predominantly belonging to the WNT subtype (*p* = 1 × 10^−4^) and showing an increased proportion of tumors sampled from the ventricle area (*p* = 0.039) (Table 1).

### 3.4. WNT-LEF1 Gene Signature Accurately Predicts WNT Subtype in Medulloblastoma Transcriptomes

**Figure 6:**
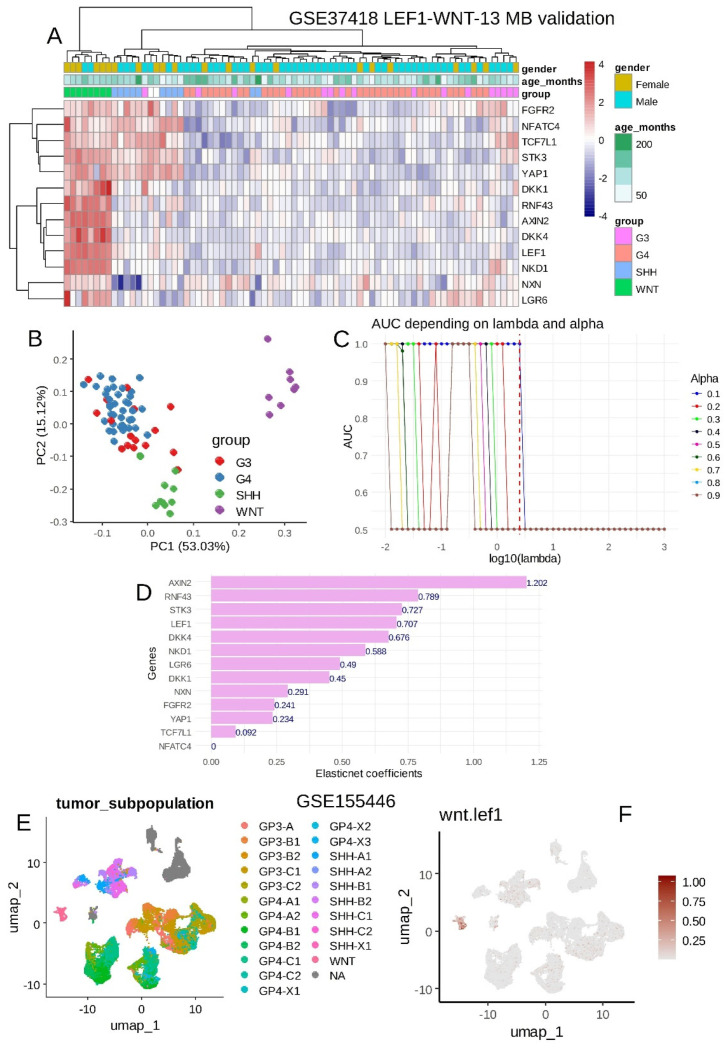
Validation of the WNT-LEF1 signature in independent medulloblastoma cohorts: GSE37418 and scRNA-seq GSE155446 datasets. (**A**) Unsupervised clustering (Euclidean distances) based on the expression of the WNT-LEF1-dependent program (GSE37418). (**B**) Principal component analysis (PCA) of the WNT-LEF1-dependent program (GSE37418). (**C**) ElasticNet tuning of lambda and alpha parameters to predict the WNT subgroup using the WNT-LEF1-dependent program. (**D**) Barplot of ElasticNet coefficients predicting the WNT subtype in the GSE37418 cohort. (**E**) WNT-MB subtypes in the single-cell transcriptome dataset GSE155446 (28 MB tumor samples). (**F**) WNT-LEF1-dependent score in the single-cell RNA-seq dataset GSE155446.

For the validation of the 13-gene signature, an independent medulloblastoma transcriptome cohort (GSE37418; Table 2) was analyzed. This cohort included 74 tumor samples, with most patients being male (72%) and a mean age at a sampling of 99 months. Unsupervised clustering based on the expression of the 13 WNT-LEF1 genes effectively stratified WNT samples from other medulloblastoma subtypes (Euclidean distances and the Ward.D2 method; Figure 6A). This stratification was further confirmed using unsupervised principal component analysis (Figure 6B).

Supervised machine learning tuned using ElasticNet regression (alpha = 0.1; Figure 6C and Supplemental Figure S7a,b) allowed us to predict WNT-subtype status with a perfect area under the curve (AUC) of 1.00 (Figure 6C). ElasticNet coefficients (Figure 6D) highlighted the predictive importance of genes such as AXIN1, RNF43, STK3, LEF1, and DKK1 for identifying the WNT subtype status in this medulloblastoma validation cohort.

A WNT-LEF1 expression score was computed for this validation cohort, identifying 8 samples with high WNT-LEF1 expression scores and 66 samples with low scores (Table 2). In this cohort, patients with high WNT-LEF1 expression scores were significantly more likely to be female (*p* = 0.007) and perfectly matched the WNT subtype (Table 2 and Supplemental Figure S7c). The ROC curve analysis of the combined expression of these 13 WNT-related LEF1 target genes confirmed their ability to predict the WNT subtype in medulloblastoma, achieving an AUC of 1.00 with 100% specificity and 100% sensitivity in the PBTA medulloblastoma cohort (Supplemental Figure S7d).

Finally, to investigate the single-cell heterogeneity of the signature in medulloblastoma cells, scRNA-seq experiments from 28 medulloblastoma tumors representing the four distinct subtypes (GSE155446; Figure 6E) were analyzed for the expression of the 13 WNT-LEF1 genes. The single-cell WNT-LEF1 expression score was found to be positive exclusively in the cluster of WNT tumor cells (Figure 6F). At the single-cell level in medulloblastoma, the WNT-LEF1 13-gene signature was expressed only in tumor cells from the WNT subtype.

### 3.5. Single-Cell Analysis Validates WNT-LEF1 Signature Activation in Hepatoblastoma Tumor Cells

**Figure 7:**
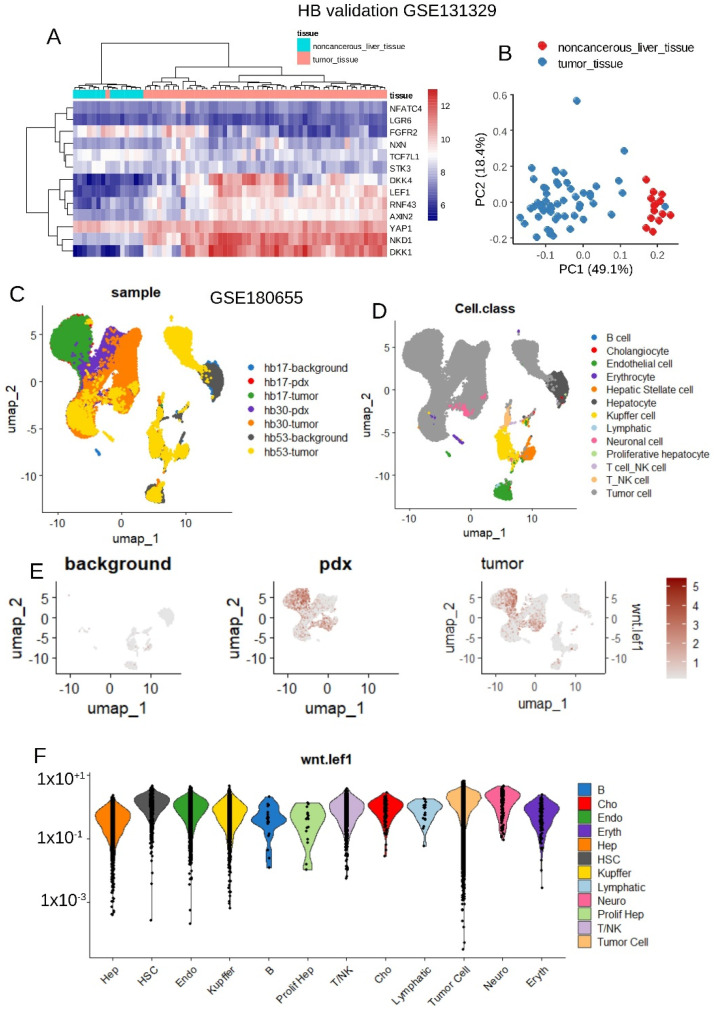
Validation of the WNT-LEF1 signature in an independent hepatoblastoma cohort: GSE131329 and scRNA-seq GSE180655 datasets. (**A**) Unsupervised clustering (Euclidean distances) based on the expression of the WNT-LEF1 program (GSE131329). (**B**) Principal component analysis (PCA) of the WNT-LEF1 program (GSE131329). (**C**) UMAP dimensionality reduction showing sample identities in the scRNA-seq dataset GSE180655. (**D**) UMAP dimensionality reduction showing cell-type identities in the scRNA-seq dataset GSE180655. (**E**) WNT-LEF1-dependent score in the single-cell RNA-seq dataset GSE180655, stratified by sample types (background: adjacent normal liver; pdx: patient-derived xenotransplantation model; tumor: HB tumor samples). (**F**) WNT-LEF1-dependent score in the single-cell RNA-seq dataset GSE180655, stratified by cell types.

Like medulloblastoma, hepatoblastoma is also of embryonic origin, and tumor cells are frequently affected by genomic alterations in the *CTNNB1* gene [52]. The expression of the 13 genes from the WNT-LEF1 signature was analyzed in the hepatoblastoma transcriptome cohort GSE131329 (Table 3), which includes 14 noncancerous liver samples and 53 hepatoblastoma tumors. Unsupervised clustering based on the expression of the 13-gene signature effectively stratified hepatoblastoma tumor samples from noncancerous liver tissue (Euclidean distances and the Ward.D2 method; Figure 7A). This stratification was further validated by unsupervised principal component analysis (Figure 7B).

Notably, more than 70% of tumors in this cohort exhibited genetic alterations in the *CTNNB1* gene (Table 3). To identify the specific cell types in which the WNT-LEF1 signature was activated, single-cell transcriptome data from human and PDX hepatoblastoma samples, compared to normal adjacent liver tissue, were analyzed (GSE180655; Figure 7C). Thirteen distinct cell types were characterized in these samples (Figure 7D).

The WNT-LEF1 single-cell expression score was computed for cells in these experiments. The score was negative in normal adjacent liver tissue but positive in PDX and tumor samples (Figure 7E). This score was confirmed to be significantly higher in tumor cells (Figure 7F and Table 4). At the single-cell level, the expression of the WNT-LEF1 13-gene signature was confirmed to be activated in tumor cells from human hepatoblastoma.

## 4. Discussion

The WNT signaling pathway plays a crucial role in the tumorigenesis of both medulloblastoma and hepatoblastoma, with organ-specific genetic alterations contributing to their development. In medulloblastoma, particularly the WNT subgroup, mutations in *CTNNB1* (β-catenin) are prevalent, leading to aberrant pathway activation [8]. Conversely, in hepatoblastoma, WNT pathway dysregulation often arises from mutations in *CTNNB1* [7] as well as deletions or the epigenetic silencing of *AXIN1* and *AXIN2* [53], which act as negative regulators of the pathway. These organ-specific genetic changes underscore the importance of WNT signaling in the pathogenesis of these pediatric tumors, providing a foundation for targeted therapeutic approaches. During this study, the transcription factor LEF1 was found to be commonly upregulated in hepatoblastoma and medulloblastoma tumors. While LEF1 deregulation in hepatoblastoma remains poorly understood, in the PBTA medulloblastoma cohort, high LEF1 expression was specifically associated with the WNT subtype. Patients with WNT-subtype medulloblastoma generally exhibit better prognoses than those with other subtypes [54]. LEF1 immunohistochemistry has been recognized as a more reliable diagnostic biomarker than β-catenin for identifying WNT-activated medulloblastoma subtypes [55,56]. Additionally, LEF1, together with TCF3, has been implicated in maintaining the self-renewal of mouse embryonic stem cells [57].

An integrative analysis of WNT-LEF1 targets in the PBTA medulloblastoma cohort identified 141 LEF1-activated genes specific to the WNT subtype. Functional enrichment revealed that 13 of these genes were involved in WNT pathway regulation, while others were mainly associated with mesenchymal development and extracellular matrix organization. LEF1 is known to facilitate epithelial–mesenchymal transition (EMT), a hallmark of cancer progression characterized by increased migration and invasion of tumor cells [58,59].

Among LEF1 targets, MYCN was identified as a direct target through promoter binding. During embryonic development, the MYCN promoter exhibits the H3K27-acetylation histone mark, indicating active chromatin at this stage. MYCN is a proto-oncogene encoding a basic helix–loop–helix transcription factor that is upregulated in hepatoblastoma and other pediatric liver tumors. It plays a critical role in promoting hepatoblastoma cell proliferation [60]. In addition, recent studies in medulloblastoma have shown that, although not the most commonly used marker, the genomic amplification of MYCN can be used during diagnosis [61].

Wnt signaling is a key developmental pathway crucial for embryonic development and adult stem cell maintenance, and its dysregulation is implicated in numerous diseases. Through multi-omics analyses, we identified a WNT-LEF1 integrative signature that accurately predicts the WNT subtype of both medulloblastoma and hepatoblastoma tumors. This signature includes RNF43 (ring finger protein 43), LEF1 (lymphoid enhancer binding factor 1), NKD1 (NKD inhibitor of WNT signaling pathway 1), AXIN2 (axin 2), DKK4 (dickkopf WNT signaling pathway inhibitor 4), DKK1 (dickkopf WNT signaling pathway inhibitor 1), LGR6 (leucine-rich repeat-containing G protein-coupled receptor 6), FGFR2 (fibroblast growth factor receptor 2), NXN (nucleoredoxin), TCF7L1 (transcription factor 7 like 1), STK3 (serine/threonine kinase 3), YAP1 (Yes1 associated transcriptional regulator), and NFATC4 (nuclear factor of activated T cells 4).

RNF43 is a transmembrane E3 ligase that removes Wnt receptors from the stem cell surface and acts as a negative feedback regulator of the WNT pathway [62]. AXIN2 and NKD1 are also negative feedback regulators in Wnt signaling, with AXIN2 destabilizing cytoplasmic β-catenin and NKD1 inhibiting its nuclear localization [63]. Among the five Wnt antagonists identified, DKK4 binds to lipoprotein-receptor-related protein 5/6 (LRP5/6) and Kremen, inducing LRP endocytosis to block β-catenin signal transduction [64]. Similarly, DKK1 promotes LRP6 internalization and degradation when forming a ternary complex with Kremen and LRP6, effectively inhibiting Wnt signaling [65].

LGR6, considered a stem cell marker in various normal tissues, is associated with tissue development, regeneration, and repair. It has also been linked to the initiation and progression of certain cancers [66]. Germline mutations in FGFR2 have been described in medulloblastoma [67]. NXN (nucleoredoxin), a redox regulator of disheveled proteins, modulates WNT signaling, and its knockout in neuroblastoma cells affects self-renewal [68]. Canonical Wnt signaling safeguards naïve pluripotency during embryogenesis, with TCF7L1 playing a repressive role during primitive endoderm induction [69]. STK3 (alias MST-2) functions within the Hippo pathway during embryogenesis [70] and interacts with WNT and Notch pathways in liver carcinogenesis [71].

YAP and TAZ, transcriptional co-activators, are essential regulators of organ size and tissue homeostasis. Their dysregulation contributes to cancer, with alternative Wnt signaling pathways (Wnt5a/b and Wnt3a) activating YAP/TAZ independently of canonical β-catenin signaling. YAP/TAZ mediate alternative Wnt signaling effects, including the antagonism of β-catenin-dependent pathways [72]. Notably, the maintenance of undifferentiated embryonic stem cells by β-catenin is inversely correlated with YAP/TAZ activity [73].

In addition to the genes mentioned above, we have observed other upregulated transcription factors. HMGA2, a chromatin remodeling protein, is known to promote cellular plasticity and stemness, potentially synergizing with LEF1-driven WNT activation to enhance tumor aggressiveness [74]. Similarly, TBX2, which regulates cell-cycle checkpoints by suppressing senescence pathways, may complement LEF1 by supporting uncontrolled proliferation [75]. CREB3L2, involved in the unfolded protein response, likely contributes to tumor survival under stress, enabling cancer cells to adapt to challenging microenvironments [76]. Together, these TFs interact in oncogenic networks where LEF1 serves as a central effector of WNT signaling, underscoring the intricate transcriptional landscape driving tumorigenesis in hepatoblastoma and medulloblastoma.

## 5. Conclusions

Our findings emphasize the potential utility of the 13-gene WNT-LEF1 signature (RNF43, LEF1, NKD1, AXIN2, DKK4, DKK1, LGR6, FGFR2, NXN, TCF7L1, STK3, YAP1, and NFATC4) in distinguishing WNT-activated medulloblastoma subtypes. Previous studies have established the WNT-activated subtype as having a distinct molecular profile with a favorable prognosis compared to other subtypes. Our study provides additional granularity by highlighting LEF1-driven WNT pathway activation as a central oncogenic program. This finding reinforces the subtype-specific molecular characterization of medulloblastoma, offering potential diagnostic markers to confirm WNT pathway activation and differentiate it from non-WNT subtypes. While the prognostic significance of the WNT-activated medulloblastoma subtype is well documented (notably, its excellent survival rates with reduced treatment intensities), our findings extend this narrative by identifying specific downstream effectors of the WNT pathway. For hepatoblastoma, our study sheds light on shared oncogenic mechanisms with medulloblastoma, which may guide future investigations into prognostic stratification based on WNT-LEF1 pathway activation. We recognize the importance of avoiding overgeneralizations. While the 13-gene signature holds potential for aiding in diagnosis and understanding oncogenic mechanisms, we have framed these conclusions as hypothesis-generating and supportive of further research rather than definitive diagnostic or prognostic tools. We have emphasized the exploratory nature of these findings and their value as a foundation for subsequent validation studies in independent cohorts.

## Figures and Tables

**Table 1:** WNT-LEF1 score stratification for PBTA medulloblastoma cohort. Low and high group stratifications were performed on threshold of WNT-LEF1 expression score (high: patient with high wnt expression score values; low: patients with low wnt expression score values). *p*-values were computed by chi square tests.

Variable	Level	High (n = 21)	Low (n = 233)	Total (n = 254)	*p*-Value
SEX	Male	13 (61.9)	142 (60.9)	155 (61.0)	
	Female	8 (38.1)	91 (39.1)	99 (39.0)	1
TUMOR TYPE	Primary	20 (95.2)	135 (57.9)	155 (61.0)	
	Progression	0 (0.0)	3 (1.3)	3 (1.2)	
	Metastatic	1 (4.8)	83 (35.6)	84 (33.1)	
	Recurrence	0 (0.0)	11 (4.7)	11 (4.3)	
	Deceased	0 (0.0)	1 (0.4)	1 (0.4)	0.02348
group	WNT	21 (100.0)	0 (0.0)	21 (8.3)	
	G3	0 (0.0)	60 (25.8)	60 (23.6)	
	G4	0 (0.0)	99 (42.5)	99 (39.0)	
	SHH	0 (0.0)	74 (31.8)	74 (29.1)	<1 × 10^−4^
CNS_REGION	Ventricles *	5 (23.8)	12 (5.2)	17 (6.7)	
	Mixed	3 (14.3)	63 (27.2)	66 (26.1)	
	Posterior fossa	13 (61.9)	152 (65.5)	165 (65.2)	
	Spine	0 (0.0)	1 (0.4)	1 (0.4)	
	Hemispheric	0 (0.0)	3 (1.3)	3 (1.2)	
	Other	0 (0.0)	1 (0.4)	1 (0.4)	0.03963
	Missing	0	1	1	
EFS_STATUS	1: Recurrence	1 (4.8)	36 (15.5)	37 (14.6)	
	0: No event	19 (90.5)	121 (51.9)	140 (55.1)	
	1: Progressive—metastatic	1 (4.8)	33 (14.2)	34 (13.4)	
	1: Deceased—due to disease	0 (0.0)	7 (3.0)	7 (2.8)	
	1: Recurrence—metastatic	0 (0.0)	23 (9.9)	23 (9.1)	
	1: Second malignancy	0 (0.0)	6 (2.6)	6 (2.4)	
	1: Progressive	0 (0.0)	7 (3.0)	7 (2.8)	0.06538

* It is possible to have a medulloblastoma located in the central nervous system region, and this potentially includes the cerebral ventricles. Medulloblastoma is a brain tumor that usually originates in the cerebellum, but it can also spread to other parts of the brain, including the ventricles [16].

**Table 2:** WNT-LEF1 score stratification for GSE37418 medulloblastoma validation cohort. *p*-values were computed by chi square tests between samples with low and high values of WNT-LEF1 expression scores and by *t*-test for quantitative variables.

Variable	Level	Low (n = 66)	High (n = 8)	Total (n = 74)	*p*-Value
gender	Male	51 (77.3)	2 (25.0)	53 (71.6)	
	Female	15 (22.7)	6 (75.0)	21 (28.4)	0.00732
age_months	Mean (sd)	98.4 (38.7)	104.5 (17)	99.1 (37)	0.66172
anapath	CL	43 (65.2)	8 (100.0)	51 (68.9)	
	DN	6 (9.1)	0 (0.0)	6 (8.1)	
	AN	17 (25.8)	0 (0.0)	17 (23.0)	0.13231
ethnicity	White	38 (57.6)	4 (50.0)	42 (56.8)	
	Black	5 (7.6)	0 (0.0)	5 (6.8)	
	Asian	5 (7.6)	1 (12.5)	6 (8.1)	
	Other	2 (3.0)	1 (12.5)	3 (4.1)	
	Hispanic	12 (18.2)	1 (12.5)	13 (17.6)	
	Asian Indian	4 (6.1)	0 (0.0)	4 (5.4)	
	Pacific Islander	0 (0.0)	1 (12.5)	1 (1.4)	0.07854
group	G4	39 (59.1)	0 (0.0)	39 (52.7)	
	WNT	0 (0.0)	8 (100.0)	8 (10.8)	
	SHH	11 (16.7)	0 (0.0)	11 (14.9)	
	G3	16 (24.2)	0 (0.0)	16 (21.6)	<1 × 10^−4^

**Table 3:** Cohort description for hepatoblastoma validation cohort GSE131329. *p*-values were computed by chi square tests between sample categories and by *t* test for quantitative variables.

Variable	Level	Tumor Tissue (n = 53)	Noncancerous Liver Tissue (n = 14)	Total (n = 67)	*p*-Value
gender	Female	25 (47.2)	8 (57.1)	33 (49.3)	
	Male	28 (52.8)	6 (42.9)	34 (50.7)	0.716363
Histological type	Well_differentiated	30 (56.6)	14 (100.0)	44 (65.7)	
	Other	2 (3.8)	0 (0.0)	2 (3.0)	
	Poorly_differentiated	21 (39.6)	0 (0.0)	21 (31.3)	0.009797
age_months	Mean (sd)	27.2 (24.1)	27.6 (26.4)	27.3 (24.4)	0.964822
pretext_stage	P3	18 (34.0)	0 (0.0)	18 (34.0)	
	P2	15 (28.3)	0 (0.0)	15 (28.3)	
	P4	11 (20.8)	0 (0.0)	11 (20.8)	
	P1	9 (17.0)	0 (0.0)	9 (17.0)	NA
chic_risk stratification	Standard	31 (58.5)	0 (0.0)	31 (58.5)	
	High	14 (26.4)	0 (0.0)	14 (26.4)	
	Intermediate	8 (15.1)	0 (0.0)	8 (15.1)	NA
ctnnbi_gene alteration	Deletion	23 (43.4)	0 (0.0)	23 (43.4)	
	Wild_Type	14 (26.4)	0 (0.0)	14 (26.4)	
	Mutation (exon3)	16 (30.2)	0 (0.0)	16 (30.2)	NA
clinical_course	Alive	38 (71.7)	0 (0.0)	38 (71.7)	
	Dead	15 (28.3)	0 (0.0)	15 (28.3)	NA

**Table 4:** Cell type comparison for WNT-LEF1 score in single-cell transcriptome of hepatoblastoma. *p*-values between cell types were obtained by 2-sided *t* test.

Comparison	Mean Groupe1	Mean Groupe2	*p*_Value
Hepatocyte vs. Tumor cell	−1.088	0.45	0
Hepatic stellate cell vs. Tumor cell	0.168	0.45	2.93 × 10^−7^
Endothelial cell vs. Tumor cell	−0.264	0.45	0
Kupffer cell vs. Tumor cell	−0.686	0.45	0
B cell vs. Tumor cell	−0.628	0.45	1.24 × 10^5^
Proliferative hepatocyte vs. Tumor cell	−1.27	0.45	2.40 × 10^−24^
T cell–NK cell vs. Tumor cell	−0.652	0.45	9.86 × 10^−91^
Cholangiocyte vs. Tumor cell	−0.208	0.45	2.26 × 10^−7^
Lymphatic vs. Tumor cell	−0.416	0.45	7.62 × 10^7^
T–NK cell vs. Tumor cell	−0.267	0.45	1.25 × 10^−5^
Neuronal cell vs. Tumor cell	0.375	0.45	4.13 × 10^−1^
Erythrocyte vs. Tumor cells	−0.619	0.45	4.20 × 10^−50^

## Data Availability

Epigenetics files generated during this study and aligned on hg38 human genome are available at the following address: https://figshare.com/articles/online_resource/bigwig_and_narrow_peaks_MACS_generated_during_study_of_MB-HB/27952287 (accessed on 11 September 2024), with DOI number: https://doi.org/10.6084/m9.figshare.27952287. Scripts used during this study are published at the following web address: https://github.com/cdesterke/scripts_MBHB_LEF1 (accessed on 24 December 2024).

## Supplementary Materials

The following supporting information can be downloaded at: https://www.mdpi.com/article/10.3390/curroncol32010035/s1, Supplemental Figures: Supplementary Figure S1. Representation of the canonical WNT pathway. Supplementary Figure S2. Combat and quantile cross-normalization of hepatoblastoma and medulloblastoma tumor transcriptomes; Supplementary Figure S3. Common differentially expressed genes in hepatoblastoma and medulloblastoma tumors; Supplementary Figure S4. Functional enrichment analysis of commonly upregulated genes in tumors (HB and MB); Supplementary Figure S5. Functional enrichment analysis of commonly downregulated genes in tumors (HB and MB); Supplementary Figure S6. WNT-LEF1-dependent signature in medulloblastoma; Supplementary Figure S7. Validation of the WNT-LEF1 13-gene signature in an independent medulloblastoma cohort: GSE37418 dataset; Supplemental Tables: Table S1. Results of LIMMA analysis between tumor and normal control tissue for the common hepatoblastoma-medulloblastoma signature; Table S2. Significant signature of differential expressed genes between tumor and normal control tissue for the common hepatoblastoma-medulloblastoma signature with identification of the transcription factors; Table S3. LEF1 binding occupancy in H1 under WNT3A stimulation for the 2039 promoter regions identified by ChIP-sequencing; Table S4. Results of LIMMA analysis comparing WNT-MB subtype to others medulloblastoma in PBTA cohort and testing expression of LEF1 targets; Table S5. Table of the integrated analysis identifying 141 LEF1 targets activated in WNT-medulloblastoma subtype of PBTA cohort. ST1-5 are available at the following address: https://github.com/cdesterke/scripts_MBHB_LEF1/tree/main/ST (accessed on 24 December 2024).
