# Supplementary material for: Single-Cell RNA Sequencing Reveals LEF1-Driven Wnt Pathway Activation as a Shared Oncogenic Program in Hepatoblastoma and Medulloblastoma
Source: Curr Oncol. 2025 Jan 9;32(1):35. doi: 10.3390/curroncol32010035 (PMC11763369; doi:10.3390/curroncol32010035)
Supplement: Supplementary file 1 [file curroncol-32-00035-s001.zip › Desterke 2024 - Sup Figs CDnew.pdf]

Current Oncology

MDPI

Article

Single-Cell RNA Sequencing Reveals LEF1-Driven Wnt Pathway Activation as a Shared Oncogenic Program in Hepatoblastoma and Medulloblastoma

Christophe Desterke[1], Yuanji Fu[2], Jenny Bonifacio-Mundaca[3], Claudia Monge[4], Pascal Pineau[4], Jorge Mata-Garrido[4,6,*] & Raquel Francés[5,6,*]

[1] Faculté de Médecine du Kremlin Bicêtre, Université Paris-Saclay, INSERM UMRS-1310, Villejuif, France; christophe.desterke@inserm.fr
[2] Université Paris Cité, INSERM, CNRS, Institut Necker Enfants Malades, F-75015, Paris, France; yuanji.fu@inserm.fr
[3] National Tumor Bank, Department of Pathology, National Institute of Neoplastic Diseases, Peru; jenny.bonifacio@upch.pe
[4] Institut Pasteur, Université Paris Cité, Unité Organisation Nucléaire et Oncogenèse, INSERM U993, Paris, France; claudia.monge@pasteur.fr; pascal.pineau@pasteur.fr
[5] Energy & Memory, Brain Plasticity Unit, CNRS, ESPCI Paris, PSL Research University, Paris, France
[6] Co-senior authors.
* Correspondence: jorge.mata-garrido@pasteur.fr; raquel.frances@espci.fr

Abstract: (1) Background: Hepatoblastoma and medulloblastoma are two types of pediatric tumors with embryonic origins. Both tumor types can exhibit genetic alterations that affect β-catenin and Wnt pathway; (2) Materials and Methods: This study used bioinformatics and integrative analysis of multi-omics data at both the tumor and single-cell levels to investigate these two distinct pediatric tumors: medulloblastoma and hepatoblastoma; (3) Results: Cross-transcriptome analysis revealed a commonly regulated expression signature between hepatoblastoma and medulloblastoma tumors. Among the commonly upregulated genes, the transcription factor LEF1 was significantly expressed in both tumor types. In medulloblastoma, LEF1 upregulation is associated with the WNT-subtype. Analysis of LEF1 genome binding occupancy in H1 embryonic stem cells identified 141 LEF1 proximal targets activated in WNT-medulloblastoma, 13 of which are involved in Wnt pathway regulation: *RNF43*, *LEF1*, *NKD1*, *AXIN2*, *DKK4*, *DKK1*, *LGR6*, *FGFR2*, *NXN*, *TCF7L1*, *STK3*, *YAP1*, and *NFATC4*. ROC curve analysis of the combined expression of these 13 WNT-related LEF1 targets yielded an area under the curve (AUC) of 1.00, indicating 100% specificity and sensitivity for predicting the WNT subtype in the PBTA medulloblastoma cohort. An expression score based on these 13 WNT-LEF1 targets accurately predicted the WNT-subtype in two independent medulloblastoma transcriptome cohorts. At the single-cell level, the WNT-LEF1 expression score was exclusively positive in WNT-medulloblastoma tumor cells. This WNT-LEF1-dependent signature was also confirmed as activated in the hepatoblastoma tumor transcriptome. At the single-cell level, the WNT-LEF1 expression score was higher in tumor cells from both human hepatoblastoma samples and a hepatoblastoma patient-derived xenotransplant model; (4) Discussion: This study uncovered a shared transcriptional activation of a LEF1-dependent embryonic program, which orchestrates the regulation of the Wnt signaling pathway in tumor cells from both hepatoblastoma and medulloblastoma.

Keywords: LEF1, WNT, pluripotency, pediatric cancer, hepatoblastoma, medulloblastoma. genome binding occupancy, scRNA sequencing, omics.

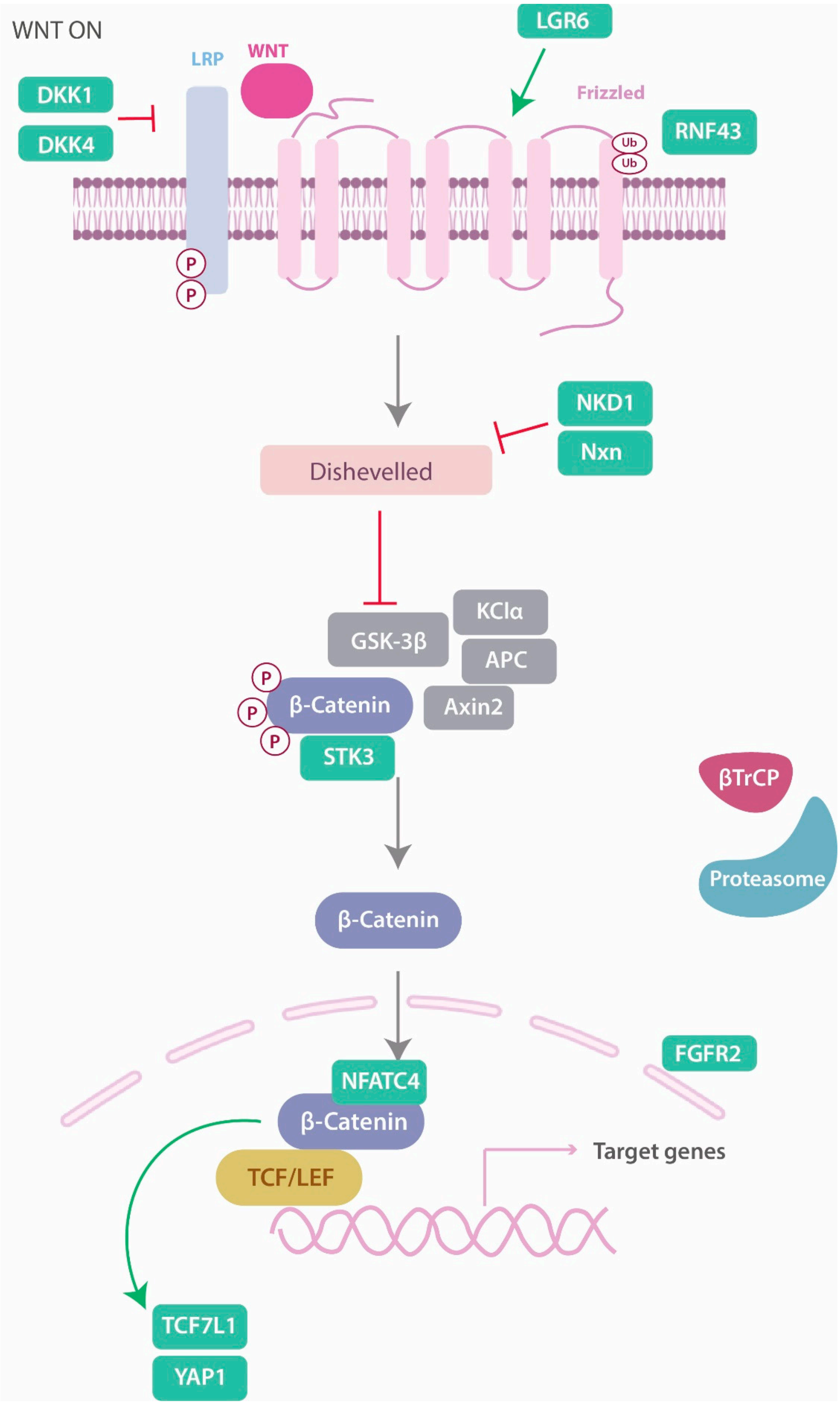

Supplementary Figure S1. Schematic representation of the canonical WNT pathway, including the genes observed in our transcriptomic analysis.

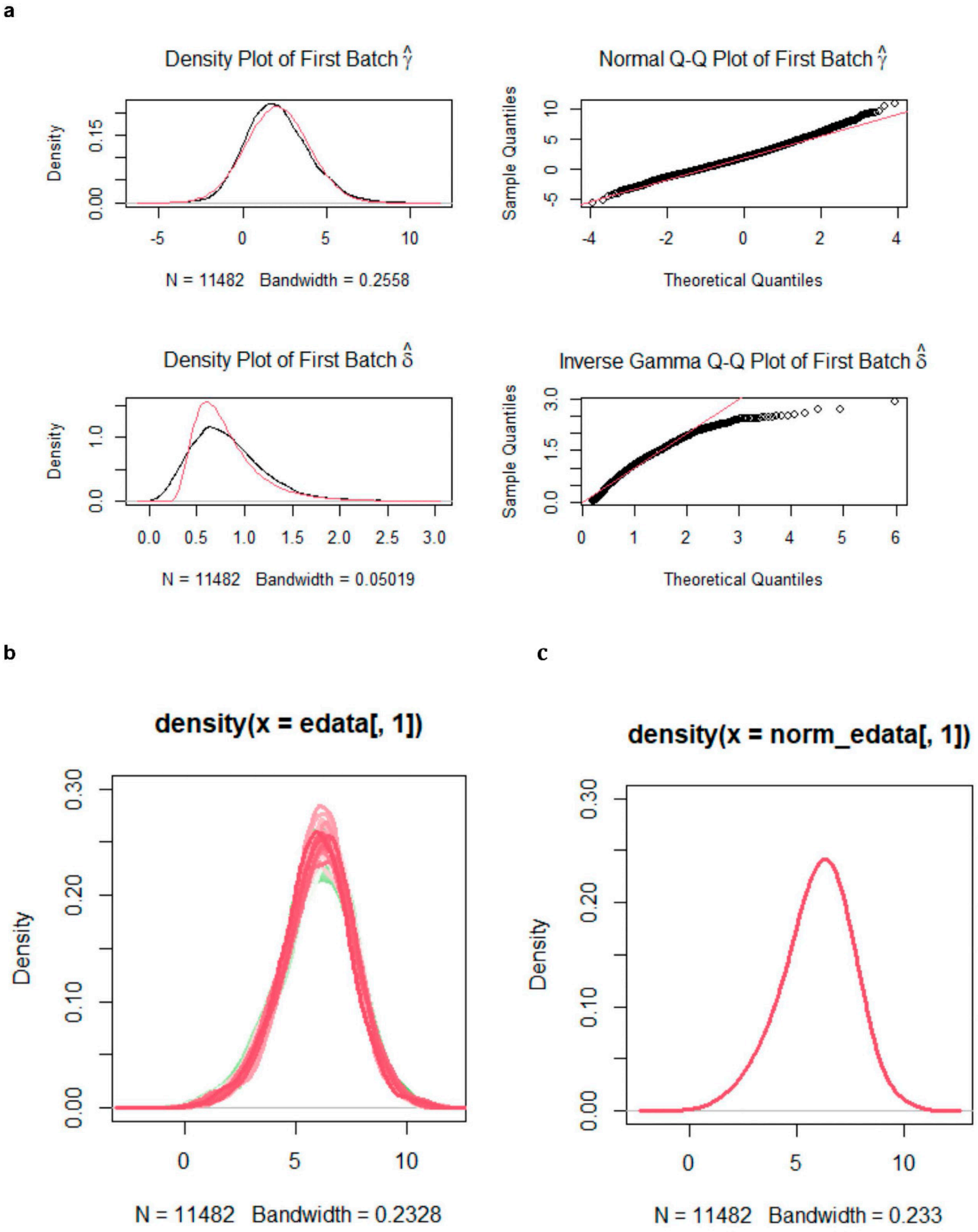

Supplementary Figure S2. Combat and quantile cross-normalization of hepatoblastoma and medulloblastoma tumor transcriptomes. (a) Combat normalization process applied to cross-normalize datasets: GSE37418 (medulloblastoma), GSE44971 (normal cerebellum), and GSE104766 (hepatoblastoma and normal liver). (b) Density plot for samples in the cross matrix after Combat normalization (11,482 common features). (c) Density plot after quantile normalization of the cross matrix.

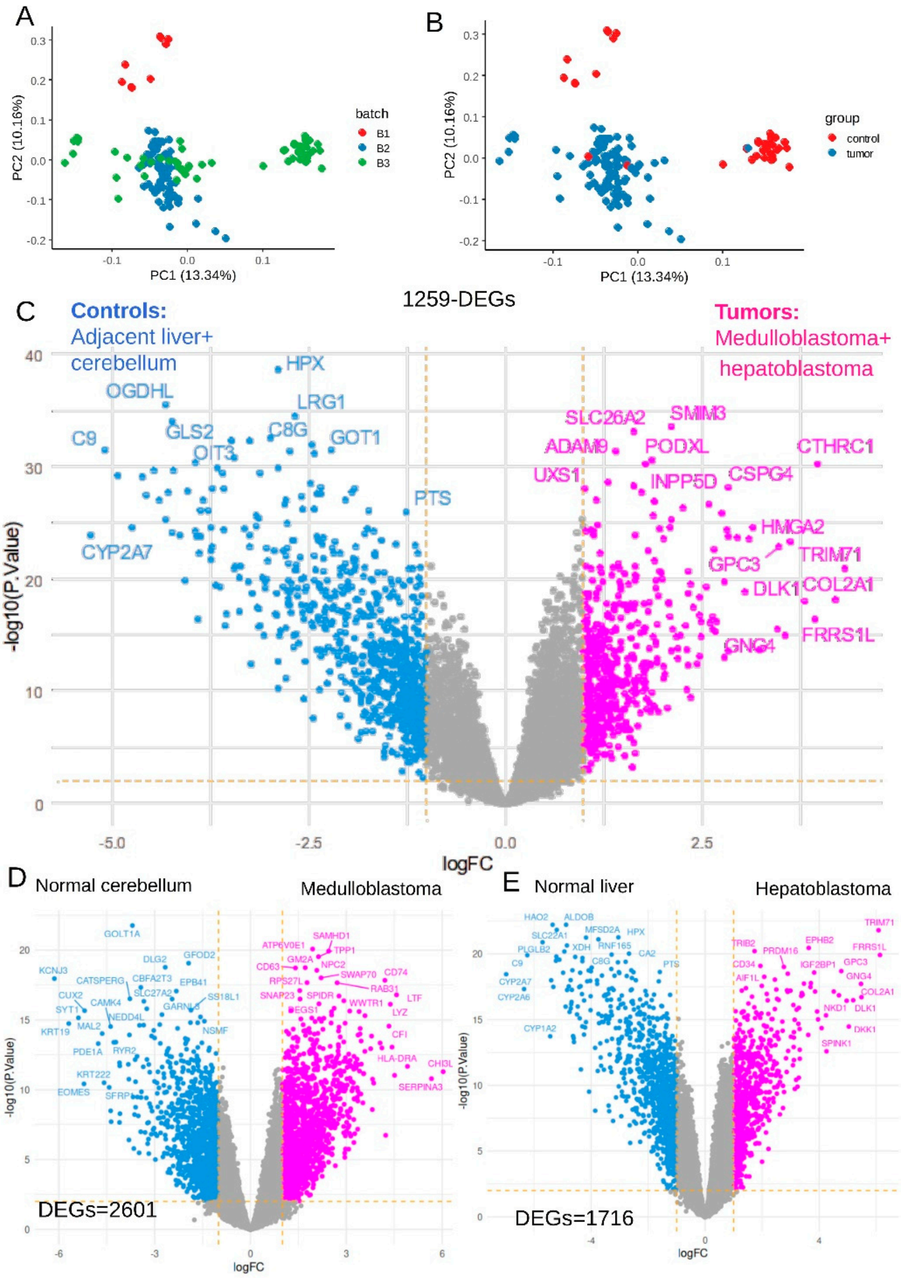

Supplementary Figure S3. Common differentially expressed genes in hepatoblastoma and medulloblastoma tumors: cross-normalized matrix. (A) Principal component analysis (PCA) of the cross matrix stratified by dataset batches. (B) Principal component analysis (PCA) of the cross matrix stratified by tissue groups (normal tissues: cerebellum and liver, versus tumors: medulloblastoma and hepatoblastoma). (C) Volcano plot of differentially expressed genes between tumors and normal tissues in the cross matrix, (D) Volcano plot of differentially expressed genes between medulloblastoma tumors and normal cerebellum samples; (E) Volcano plot of differentially expressed genes between hepatoblastoma tumors and normal liver samples.

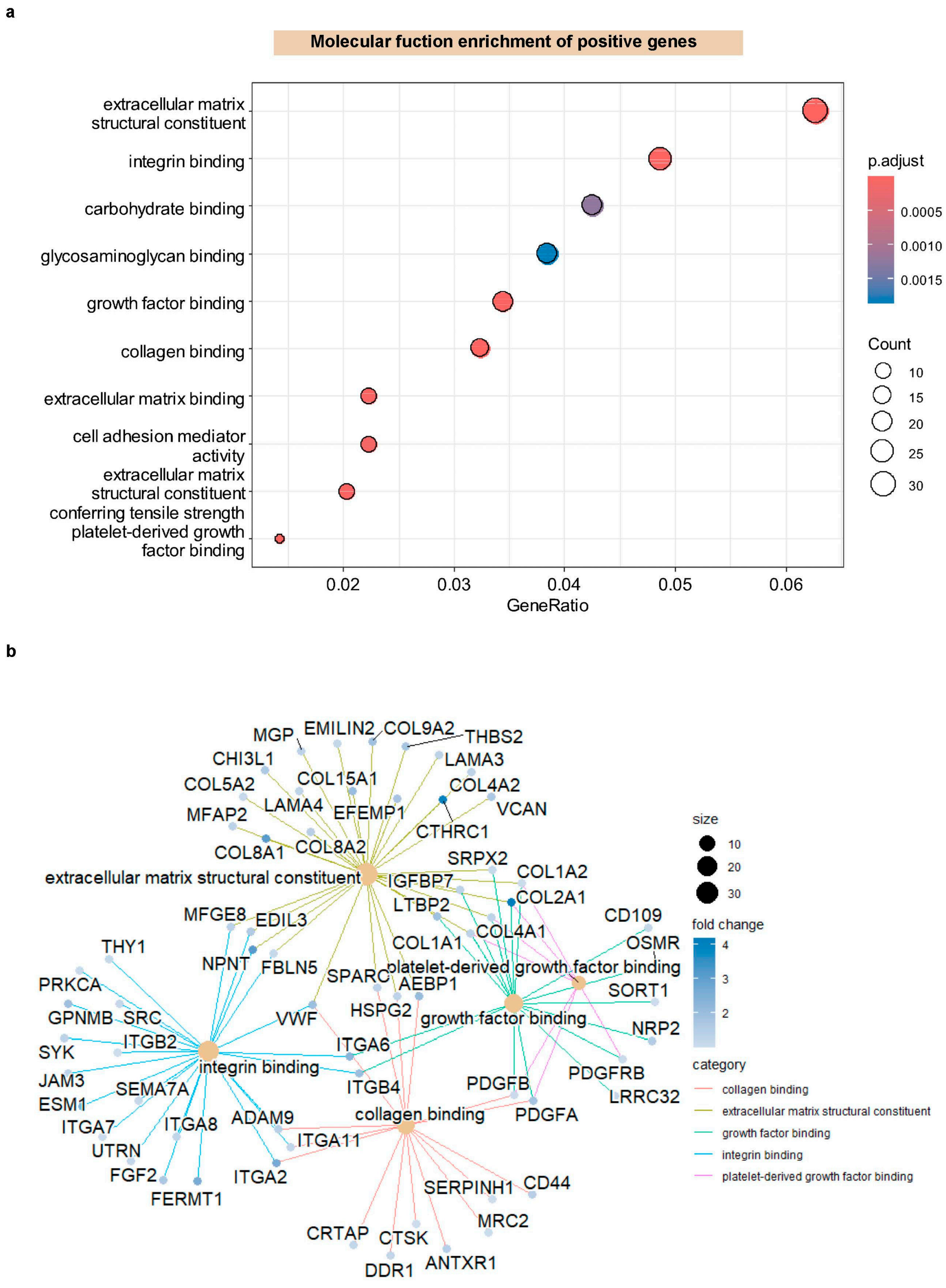

Supplementary Figure S4. Functional enrichment analysis of commonly upregulated genes in tumors (HB and MB). (a) Dot plot showing the top 10 enriched molecular functions for genes upregulated in both tumors (HB and MB). (b) Functional enrichment network illustrating upregulated genes involved in the top molecular functions.

a

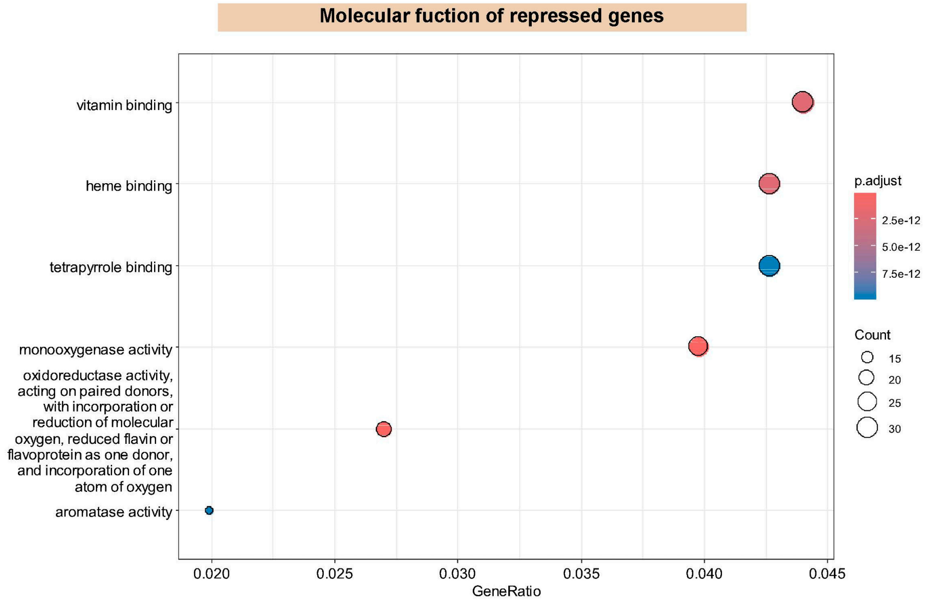

b

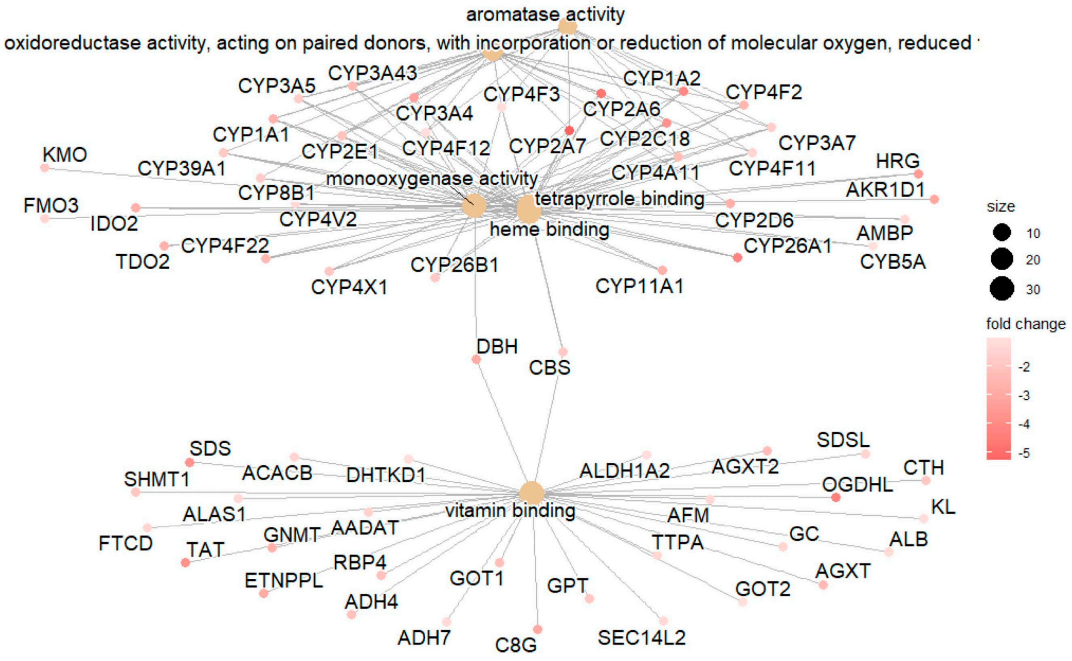

Supplementary Figure S5. Functional enrichment analysis of commonly downregulated genes in tumors (HB and MB). (a) Dot plot showing the top 10 enriched molecular functions for genes downregulated in both tumors (HB and MB). (b) Functional enrichment network illustrating downregulated genes involved in the top molecular functions.

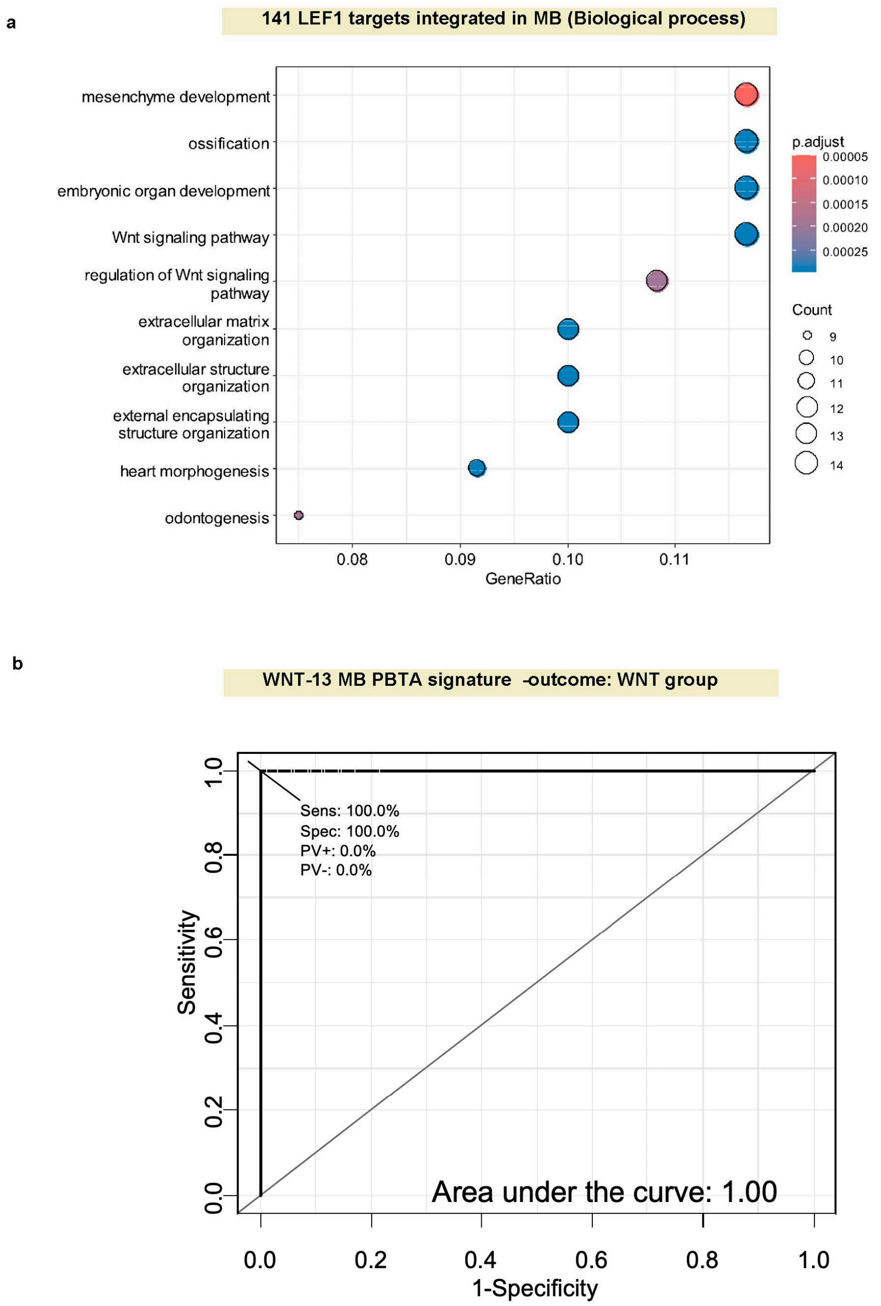

Supplementary Figure S6. WNT-LEF1-dependent signature in medulloblastoma. (a) Dot plot of functional enrichment (Gene Ontology Biological Process) for the 141 LEF1 targets identified in the WNT subtype of medulloblastoma from the PBTA cohort. (b) ROC curve demonstrating the predictive performance of the 13-gene WNT-LEF1 signature for the WNT subtype in the MB-PBTA cohort.

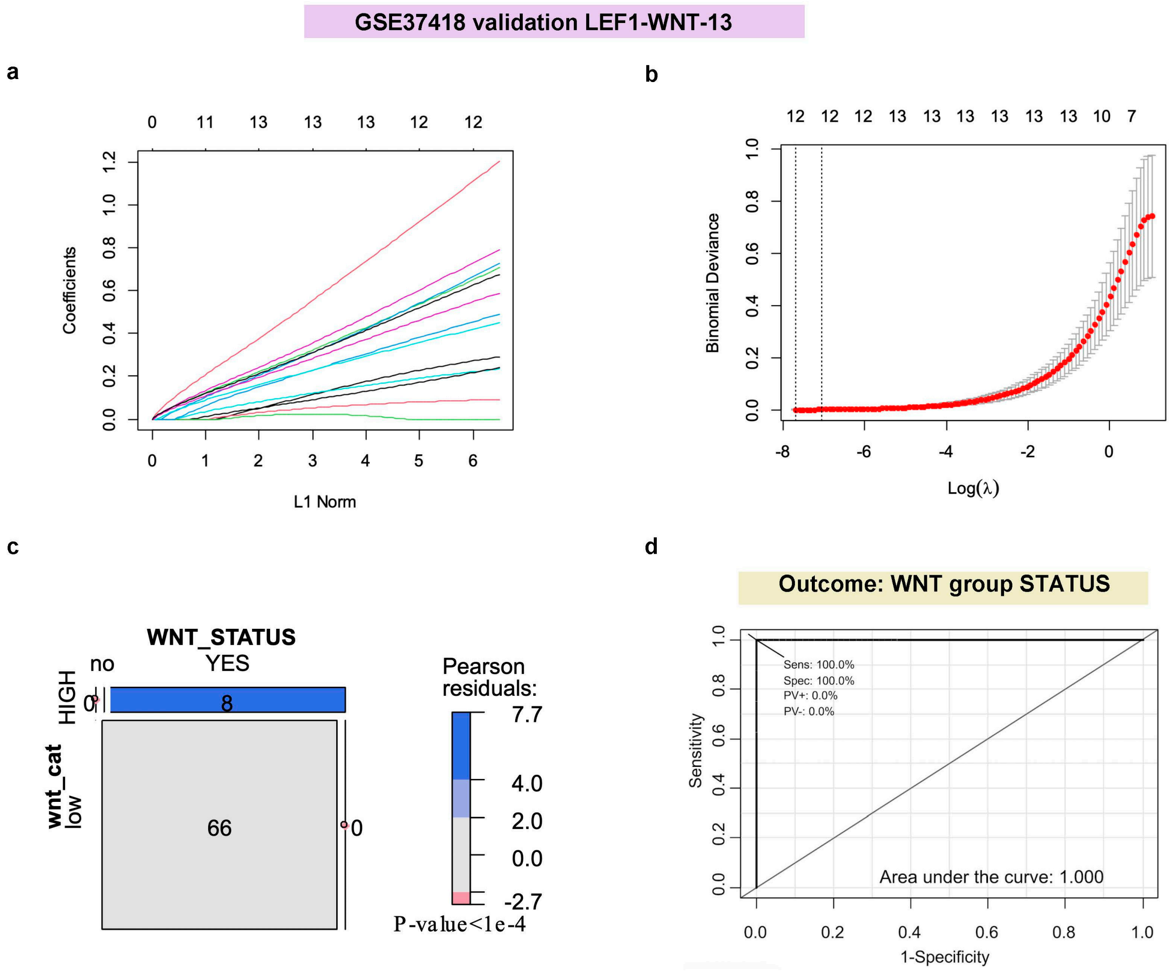

Supplementary Figure S7. Validation of the WNT-LEF1 13-gene signature in an independent medulloblastoma cohort: GSE37418 dataset. (a) ElasticNet model fitted on the 13-gene WNT-LEF1 signature to predict the WNT subtype in the MB validation cohort, with the alpha parameter fixed at 0.1. (b) Coefficient variation of the 13-gene WNT-LEF1 signature from the ElasticNet model to predict the WNT subtype in the MB validation cohort, with the alpha parameter fixed at 0.1. (c) Mosaic plot testing the association between WNT categories predicted using the LEF1-WNT expression score and the known WNT subtype status. (d) ROC curve demonstrating the predictive performance of the 13-gene WNT-LEF1 signature for the WNT subtype in the medulloblastoma validation cohort.

Supplemental Tables

Table S1. Results of LIMMA analysis between tumor and normal control tissue for the common hepatoblastoma-medulloblastoma signature

Table S2. Significant signature of differential expressed genes between tumor and normal control tissue for the common hepatoblastoma-medulloblastoma signature with identification of the transcription factors

Table S3. LEF1 binding occupancy in H1 under WNT3A stimulation for the 2039 promoter regions identified by ChIP-sequencing

Table S4. Results of LIMMA analysis comparing WNT-MB subtype to others medulloblastoma in PBTA cohort and testing expression of LEF1 targets

Table S5. Table of the integrated analysis identifying 141 LEF1 targets activated in WNT-medulloblastoma subtype of PBTA cohort. Tables S1–S5 are available at the following ad-dress: https://github.com/cdesterke/scripts_MBHB_LEF1/tree/main/ST (accessed on 24 December 2024).
